# Management of capsular rupture and vitreous loss in cataract surgery

**Published:** 2008-03

**Authors:** Nick Astbury, Mark Wood, Uday Gajiwala, Rajesh Patel, In the Sewa Rural Team, Yi Chen, Larry Benjamin, Sunday O Abuh

**Affiliations:** Consultant Ophthalmic Surgeon, Norfolk and Norwich University Hospital NHS Trust, Colney Lane, Norwich NR4 7UY, UK.; Consultant Ophthalmologist, CCBRT Hospital, Box 23310, Dar es Salaam, Tanzania. Email: markwood@cats-net.com; Sewa Rural, Jhagadia District, Bharuch, Gujarat 393 110, India.; Sewa Rural, Jhagadia District, Bharuch, Gujarat 393 110, India.; People Eye Centre, Peking University People's Hospital, Beijing 100044, China. Email: chenyi88888@vip.sina.com; Consultant Ophthalmic Surgeon, Department of Ophthalmology, Stoke Mandeville Hospital, Mandeville Road, Aylesbury, Buckinghamshire, HP21 8AL, UK.; Paediatric Ophthalmologist, ECWA Eye Hospital, PO Box 14, Kano, Nigeria. Email: absund@yahoo.com

**Figure:**
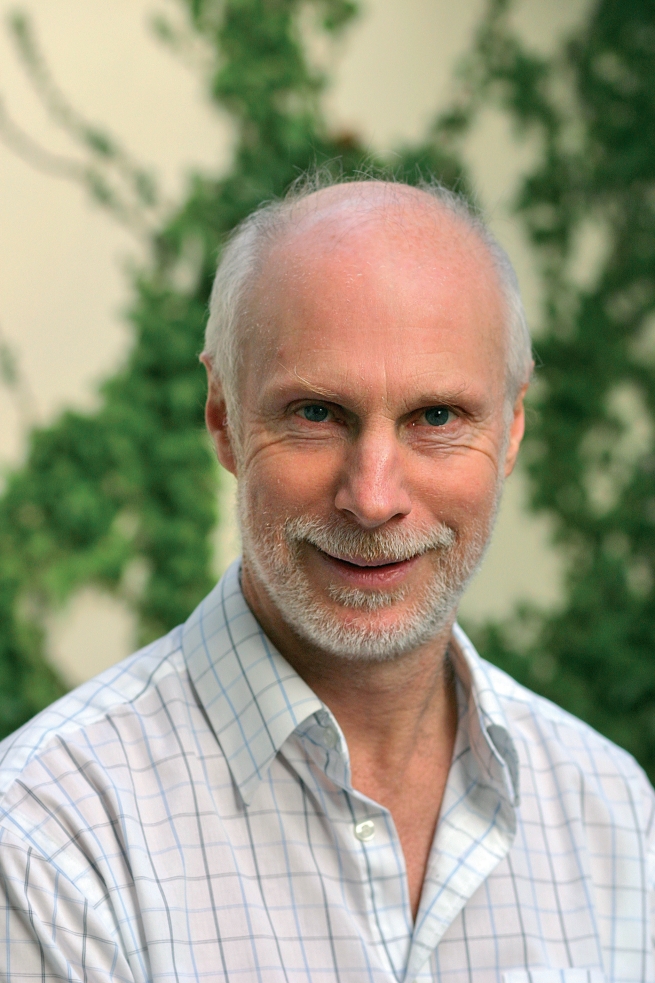
**Nick Astbury** Consultant Ophthalmic Surgeon, Norfolk and Norwich University Hospital NHS Trust, Colney Lane, Norwich NR4 7UY, UK.

Every eye surgeon has experienced – or will experience – that sinking feeling when the posterior capsule is ruptured and vitreous comes forward into the anterior chamber. At that moment everything changes, including the heart rate of the surgeon and the possible outcome for the patient.

But all is not lost. If the theatre team are well prepared, the situation can be managed calmly and professionally in order to achieve the best possible visual result.

It is most important to remove every trace of vitreous from the wound and anterior chamber. Failure to achieve this increases the risks of leakage, of infection due to a vitreous wick, or of vitreous traction that may lead to cystoid macular oedema or retinal detachment.

In an ideal world, automated vitrectomy should be the procedure of choice to deal with vitreous loss; however, if the equipment is unavailable, it may be necessary to resort to the ‘sponge and scissors’ vitrectomy method.

Implanting an intraocular lens (IOL), although desirable, should not be undertaken at any cost if it will involve further trauma to the eye.

It is worth mentioning that pressure from the speculum is often to blame for the difficulty surgeons experience in dealing with capsular rupture and vitreous loss. Therefore, it is always advisable to make sure that the speculum is not pressing on the eye.

Below, five ophthalmologists from around the world present their tips on managing this complication. Their opinions and methods differ, depending upon individual circumstances and available resources.

**Figure:**
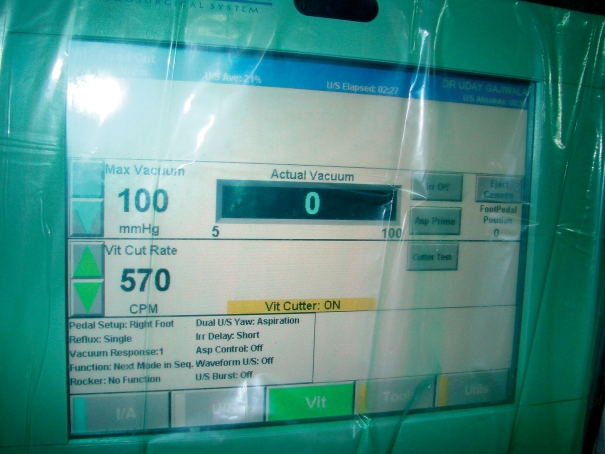
**Vitrectomy parameters set on a phaco machine used to perform automated vitrectomy after capsular rupture and vitreous loss. INDIA**

**Figure:**
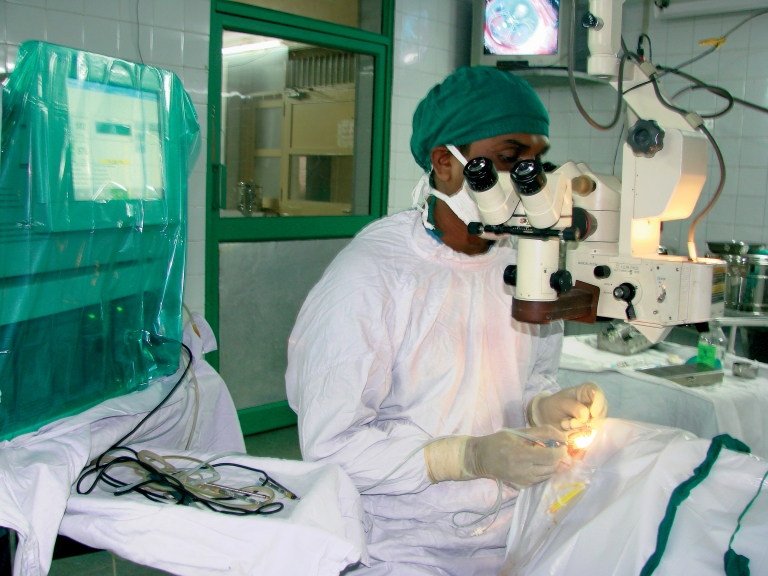
**A surgeon performs a vitrectomy after capsular rupture and vitreous loss. INDIA**

## Tanzania

**Figure:**
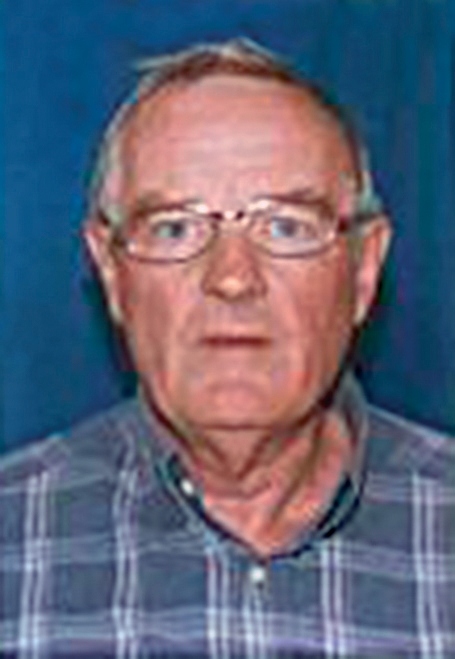
**Mark Wood** Consultant Ophthalmologist, CCBRT Hospital, Box 23310, Dar es Salaam, Tanzania. Email: markwood@cats-net.com

Capsular rupture is a dreaded complication of cataract surgery; it jeopardises the chances of inserting a posterior lens and therefore obtaining the ideal optical correction of the patient's aphakia after the operation. However, if this complication does occur, do not panic: most cases can be salvaged.

### Tip 1

**Stop everything**. Sit back and **think**. Get your vitrector ready while thinking. The Guerder Vitron anterior vitrectomy machine is ideal and should be made available to all cataract surgeons. You will have to perform an **anterior vitrectomy**. Try to preserve as much capsule as possible while you do this.

### Tip 2

After you have done a vitrectomy, if you are not sure how much capsule remains it may be wise to close the incision and consider implanting a **secondary IOL**. Later, you can use the slit lamp to visualise the remaining capsule and plan your operation.

### Tip 3

If you have done a continuous curvilinear capsulorhexis, you should be able to **insert a lens in the sulcus**, as the anterior rim of the capsule will hopefully still be there. With a linear capsulotomy this may be possible as well. I usually insert a hard lens into the sulcus and abandon any idea of using a foldable lens. If there is enough capsule inferiorly, I use an Aurolab scleral fixation lens. This IOL has the advantage of having a large optic of 6.5 mm which gives it added stability; it can be sutured with 10-0 Prolene to the iris at the 12 o'clock position through the hole in the haptic. This is not possible with small incision surgery, because you cannot suture the lens to the iris down a tunnel incision.

### Tip 4

If you have done small incision surgery, it is more difficult to manage vitreous loss. In this circumstance, I would probably close the eye and implant a secondary IOL. It is always preferable to implant a posterior lens. However, if this is not possible, an anterior chamber lens is a good alternative. Do not forget to do an **iridectomy**. I perform two iridectomies when placing an anterior lens.

### Tip 5

In a patient with an only eye, do not forget that +10 **aphakic correction spectacles** can give good vision; this is better than struggling to insert an imperfect IOL which may cause more damage to the tissues.

## India

**Uday Gajiwala (left), Rajesh Patel, and others in the Sewa Rural Team** Sewa Rural, Jhagadia District, Bharuch, Gujarat 393 110, India.

**Figure:**
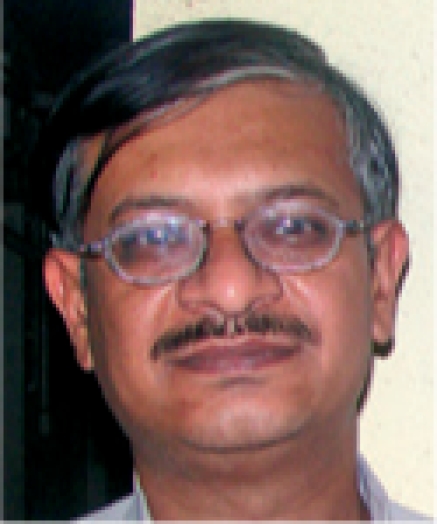


**Figure:**
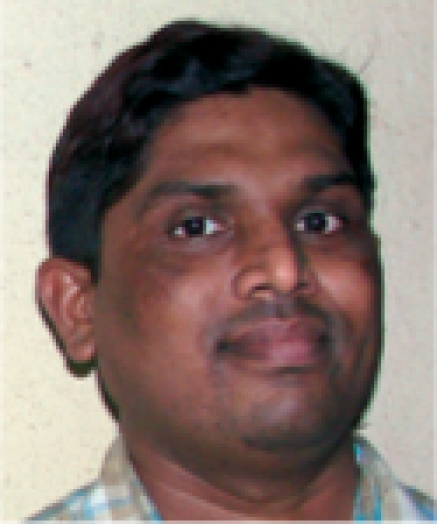


### Tip 1

If capsular rupture and vitreous loss occurs after complete removal of the lens matter, perform a good anterior vitrectomy (an automated vitrectomy probe without in-built irrigation is preferable).

To prevent extension of the tear, hydration of the vitreous, and flushing of the vitreous, **the cut rate of the vitrectomy machine should be high** (up to 800 cuts per minute) and **the vacuum should be low** (approximately 50 mmHg).

### Tip 2

If capsular rupture and vitreous loss occurs while some lens matter remains, perform a good automated **anterior vitrectomy and cortex removal**, ensuring aspiration of the cortex towards the tear and not away from it. Dry aspiration is most suitable.

### Tip 3

**Complete removal of vitreous** from the anterior chamber is indicated by a round pupil, the falling back of the iris, and the formation of a single air bubble after air injection.

### Tip 4

#### Special situations

- If the capsular tear is in the inferior position, be careful because the IOL can drop from the tear into the vitreous cavity.
- If there is a bulge in the vitreous material, giving a systemic injection of mannitol while the patient is on the operating table may help to reduce the pressure.

**Figure:**
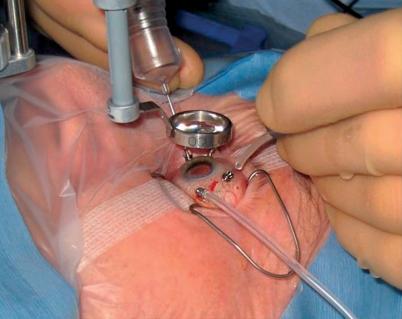
**Performing a three-port pars plana vitrectomy. UK**

### Tip 5

#### Placement of the IOL

In the case of a posterior capsular tear involving less than a third of the periphery, the IOL can be placed with the haptics positioned away from the tear; the stability of the IOL should then be checked.

With a central tear and an adequate rim all the way around, a sulcus-fixated IOL can be implanted. The IOL can be placed in front of the anterior capsule if the rhexis is round and the rim is adequate in size. But if the support is less than adequate, it may be necessary to place an anterior chamber IOL or a sclerally fixated IOL.

In all cases of posterior capsular tear, it is important to use an IOL with a large optic size (>6.0 mm) and with a large overall diameter (>13.5 mm).

Always remember:

- The importance of recognising the complication as early as possible cannot be overemphasised.
- Do not panic, keep calm, keep to the basic rules.
- No vitreous should be left in the iris plane or in front of it.
- ‘Sponge and scissors’ vitrectomy should be avoided if at all possible.
- The placement of the IOL depends on the capsular support available – even if the anterior capsule is available, a posterior chamber IOL can be implanted in the sulcus.
- Try to do a primary IOL implant, whether it is an anterior chamber IOL or a sclerally fixated IOL.
- It is good practice to inform the patient of the complication.

## China

**Figure:**
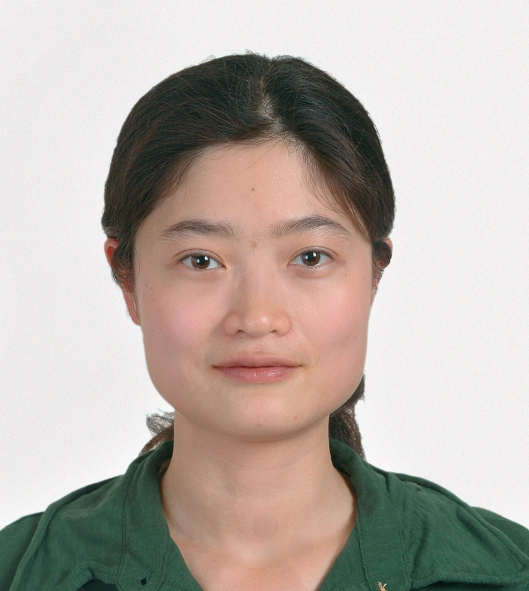
**Yi Chen** People Eye Centre, Peking University People's Hospital, Beijing 100044, China. Email: chenyi88888@vip.sina.com

### Tip 1

**Stop working** as soon as you sense that there is a problem. Carefully remove the instrument from the eye if possible. Remember that an abrupt shallowing of the anterior chamber may extend the tear and that abrupt removal of the instrument may lead to lens material falling into the vitreous. **Inject viscoelastic fluid** through the side-port incision, if necessary, before removing instruments from the eye.

### Tip 2

**Maintain the anterior chamber** and **stabilise the remaining lens material**. Filling the anterior chamber with viscoelastic helps to maintain the anterior chamber and may help to tamponade the anterior hyaloid face. Injecting viscoelastic below the remaining lens material may stabilise it.

### Tip 3

If possible, **remove all remaining lens material**. Enlarge the incision and extract the nucleus with a loop. If the capsular rupture is small, the irrigation-aspiration technique may be used to aspirate the remaining cortex. If the capsular rupture is large and cortex is mixed with vitreous, an anterior vitrectomy may be used to remove both the cortex and vitreous.

### Tip 4

If vitreous loss has occurred, remove all vitreous from the anterior chamber and the incision. A ‘sponge and scissors’ vitrectomy can be very useful if automated vitrectomy instruments are not available; however, it is unlikely to remove all vitreous from the anterior chamber. **Automated vitrectomy is preferable**.

### Tip 5

Implant the IOL according to the situation. ‘In the bag’ placement is ideal. If there is a large rupture of the posterior capsule, the surgeon may implant the IOL in the ciliary sulcus. Once the IOL has been placed, gradually instil a miotic. Afterwards, remove the viscoelastic with **irrigation-aspiration**. Raising the irrigation bottle is important to avoid any shallowing of the anterior chamber; this will help prevent further vitreous prolapse. You must pay careful attention. In particular, check the pupil and wounds to ensure that all vitreous has been removed. Always check that the wounds are watertight.

## United Kingdom

**Figure:**
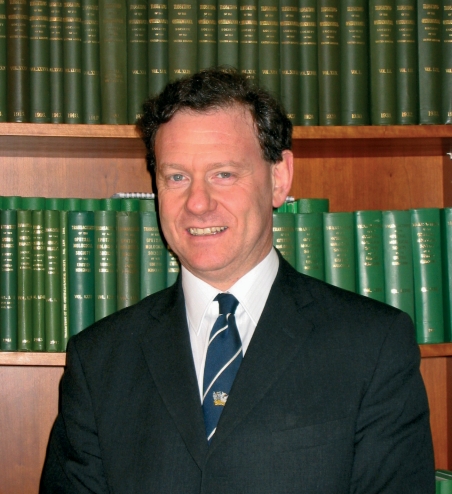
**Larry Benjamin** Consultant Ophthalmic Surgeon, Department of Ophthalmology, Stoke Mandeville Hospital, Mandeville Road, Aylesbury, Buckinghamshire, HP21 8AL, UK.

### Tip 1

If vitreous loss is managed well, the outcome can be just as good as if it had not happened. The first tip is **early recognition** that vitreous loss has taken place. The implications of vitreous loss may vary, depending on the type of cataract surgery. It tends to be a fairly expulsive event when it occurs during extracapsular surgery, but it is less so during closed-chamber phacoemulsification. By recognising the event early, steps can be taken to minimise further problems. Take time to sit back for a minute and assess the situation carefully. Do not suddenly pull instruments out of the eye – this may cause vitreoretinal traction.

### Tip 2

**Keep calm** and ask for the vitrector in a quiet, level voice (as if you were simply asking someone to pass the salt). Make sure the team environment stays calm and supportive. You should learn how to set up and use the vitrector before a case of vitreous loss has to be dealt with. This experience can be gained in a skills centre.

### Tip 3

**Figure 1:**
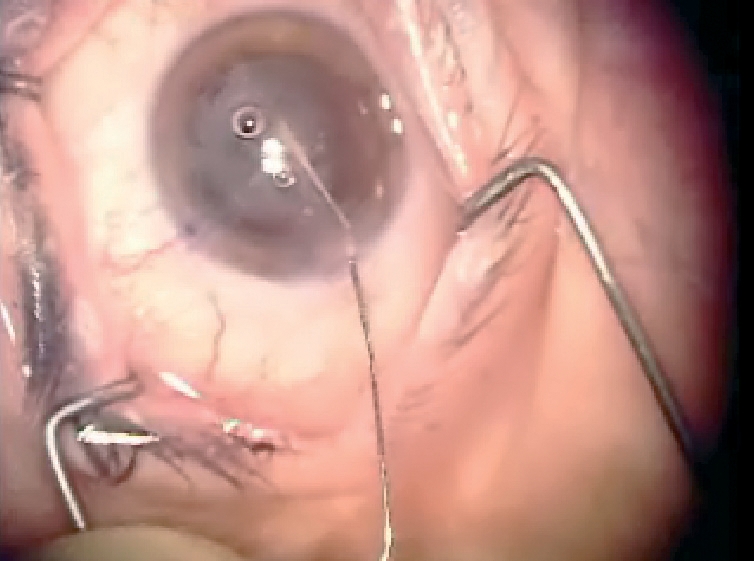
***Triamcinolone acetonide is injected into the anterior chamber to stain the vitreous and make it easier to see.***

**Figure 2:**
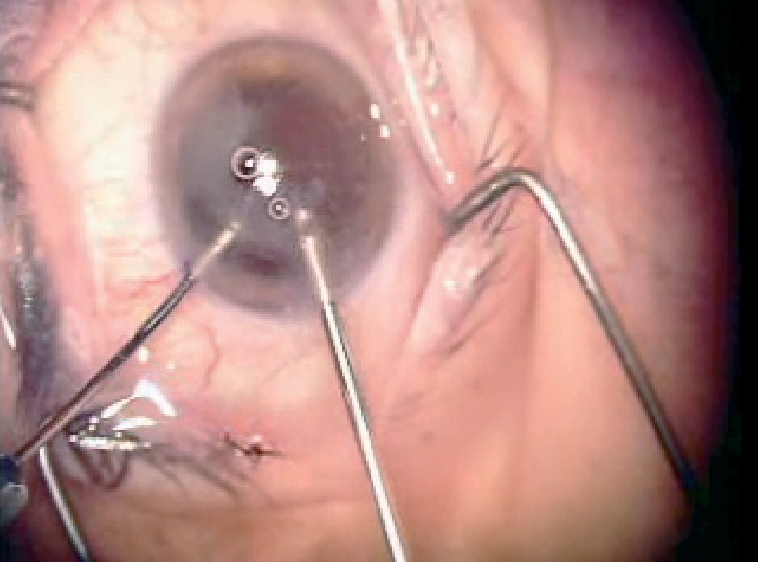
***Using a vitrector. Note the vitreous which has been stained with triamcinolone acetonide.***

Use triamcinolone acetonide (Kenelog) to **stain the vitreous** in the anterior chamber (this is an off-label use). A solution of 40 mg in 1 ml can be used for this purpose, neat or diluted twice or three times its own volume with a balanced salt solution. A gentle injection of the drug via a Rycroft cannula into the anterior chamber (Figure 1) will make the vitreous easier to see and help to guide you: you will be able to see when all the vitreous has been removed from the wound and pupillary area (Figure 2). If some triamcinolone remains in the eye, it will have anti-inflammatory properties; however, checks should be made postoperatively for a rise in intraocular pressure.

### Tip 4

Using a **separate small wound** for the vitrector prevents the main wound from becoming oedematous and maintains an effective closed chamber. Use the vitrector at the maximum cut rate (usually around 400 cuts per minute on an anterior vitrector) and make small movements of the probe within the eye. This will minimise vitreoretinal traction during the operation. It is a good idea to separate the infusion fluid from the vitrector and to start the anterior vitrectomy with no fluid running. Keep the vacuum low during this step. When the infusion is started, keep the flow rate low. An anterior chamber maintainer is a very useful device for delivering the infusion fluid. This is placed at the limbus and is self-retaining.

### Tip 5

**Postoperative follow-up** is important. The patient will need to be told that a complication has occurred and should be informed of the possible outcomes. A topical steroid, an antibiotic, and a mydriatic should be used postoperatively and regular follow-up visits should take place until the eye is quiet and further complications are ruled out or managed. If you did not place an implant during the initial operation, you can make arrangements to do this at a later date. Other forms of visual correction in the short term (such as contact lenses) can be discussed. You should carefully inspect the retinal periphery when the eye is quiet. The patient should be warned about the possible symptoms of retinal detachment, cystoid macular oedema, and infection.

## Nigeria

**Figure:**
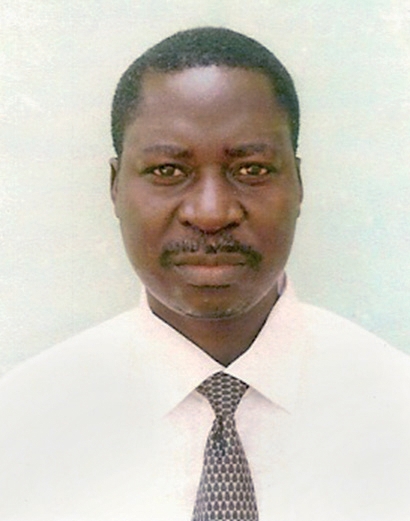
**Sunday O Abuh** Paediatric Ophthalmologist, ECWA Eye Hospital, PO Box 14, Kano, Nigeria. Email: absund@yahoo.com

### Tip 1

Vitreous loss may be inevitable in eyes that have undergone **couching** or in cases of **vitreous degeneration**. However, in all cases, vitreous loss must be carefully managed to prevent complications such as defective wound closure, high astigmatism, high or low intraocular pressure, corneal oedema from endothelial touch, retinal detachment, chronic inflammation, or cystoid macular oedema.

### Tip 2

Management starts with good **preoperative patient education** to reduce the risk of patient movement during surgery. Approximately 90–95% of our patients remain quiet during surgery without any form of premedication.

### Tip 3

Poor anaesthesia is a major contributing factor to vitreous loss. **Good anaesthesia** must achieve good pain relief (analgesia) and immobilisation (akinesia) of the lids and globe (page 14).

### Tip 4

**Avoid undue pressure on the eye** from the fixation forceps, speculum, or lens expressor. Avoid clumsy use of instruments inside the eye. Avoid creating too high a pressure when injecting the irrigating fluid into the anterior chamber.

### Tip 5

When forced to use the manual ‘sponge and scissors’ vitrectomy method:

- Work with good magnification and illumination.
- Use non-fragmenting cellulose sponges.
- Touch the vitreous in the anterior chamber with a sponge tip and cut the vitreous strands with sharp De Wecker's or Wescott's scissors.
- Avoid excessive traction on the vitreous.
- Repeat the procedure until all strands of vitreous are removed from the anterior chamber, iris surface, and wound edges.
- After removing the vitreous, sweep the iris surface with an iris repositor to check whether there is residual vitreous. If present, the pupil may be distorted. Repeat the procedure until the pupil becomes round.
- A weak solution of pilocarpine (we use four drops of 4% pilocarpine in 2 ml of normal saline solution) may be instilled into the anterior chamber to constrict the pupil and keep the vitreous behind the iris.
- Re-form the anterior chamber with air after wound closure to minimise vitreous entrapment in the wound.

